# Caregiver burden, psychological well-being, and support needs among Swedish informal caregivers

**DOI:** 10.1186/s12889-025-22074-y

**Published:** 2025-03-04

**Authors:** Sonja Togmat Malki, Peter Johansson, Gerhard Andersson, Frida Andréasson, Ghassan Mourad

**Affiliations:** 1 Department of Health, Medicine and Caring Sciences, Linköping University, Linköping, 601 74 Sweden; 2 Department of Internal Medicine in Norrköping, Vrinnevi Hospital, Norrköping, Sweden; 3 Department of Behavioural Sciences and Learning, Linköping University, Linköping, Sweden; 4 Department of Clinical Neuroscience, Karolinska Institutet, Stockholm, Sweden; 5 Department of Social Work, Linnaeus University, Kalmar, Sweden

**Keywords:** Informal caregivers, Caregiver burden, Psychological well-being, Support, Needs

## Abstract

The proportion of elderly people in the world is increasing, which increases the burden on the healthcare system and requires more formal and informal care. In Europe, informal care accounts for approximately 3.5% of GDP, and this is expected to increase. In Sweden, about 1.3 million people are informal caregivers (900,000 of whom are employed). Informal care is most common among people aged 45–65 years. Informal caregivers suffer from mental and physical health problems, such as depression, anxiety, and cardiovascular issues. The aim of this study was to describe informal caregivers in Sweden, their caregiver burden, psychological well-being, and their support needs. A web survey was used to collect data, and 379 informal caregivers responded to the survey. The results of this study showed that the majority of informal caregivers are women with good economic status and university education. Most care for one person, usually a spouse or a child, and balance work with caregiving tasks. Informal caregivers who provided care, help, and support to one person experienced greater burdens and higher levels of stress and depression. The impact on burden and psychological well-being becomes more pronounced when the informal caregiver has a close relationship or lives in the same household as the care recipient. These findings, and the fact that 82% of the participants use digital tools daily and are open to receiving support digitally, underscore the urgent need for digital interventions to reduce the caregiver burden and improve the psychological well-being of informal caregivers.

**Supplementary Information:**

The online version contains supplementary material available at 10.1186/s12889-025-22074-y.

## Introduction

The reliance on informal caregivers, defined as individuals providing care, assistance, or support to loved ones without formal training or compensation, is increasingly crucial, both on national and international levels, for example for people with physical disabilities, neurological conditions, or chronic life-limiting illnesses. This (often unpaid) care provided by family members, neighbors and friends and plays a vital role in addressing the diverse needs and challenges of care recipients, which may stem from impaired physical health, reduced function due to illness or aging, disabilities, mental health issues, dementia, or memory problems Vicente, McKee [1, 2].

With a global increase in the aging population, the needs on healthcare systems are expected to increase, which also means an increased need for the number of informal caregivers. Currently, informal care constitutes around 3.5% of total GDP in Europe, a figure projected to rise as the number of beds in health and social care decreases [3]. For instance, in Sweden alone, the cost of informal care amounts to almost SEK 13,7 billion euros per year. This includes various expenses, with the largest portion attributed to the loss of income for those caring for a loved one, accounting for 55% of the total costs. Indirect costs, quitting work, shortened working hours, reduced work capacity. Direct cost of care: length of stay, own financial costs and loss of sleep [4].

In Sweden, approximately 1.3 million people, constituting over 10% of the population, serve as caregivers, with the majority, 900,000 being employed. Caregiving is most prevalent among those aged 45–65 years, primarily helping parents. Over half of caregivers offer regular help, dedicating around 1 to 10 h per week to caregiving responsibilities. Women are more involved in supervision and personal care, while men typically provide practical and financial support [1, 5]. Despite the burden, caregiving can also be experienced as something positive. Informal caregivers often do not see themselves as caregivers; but consider it as a natural part of their relationship. This can make it hard for them to seek support [3, 6]. The situation and experiences of informal caregivers indicate that they need support emotional support in form of empathy, practical help with daily tasks, informational support, and affirmation in their situation [1].

Numerous studies highlight increased stress levels, caregiver burden, and negative emotions such as fear and low mood [7, 8]. Informal carers’ own health problems resulting from the burden of care often turn them into consumers of the health care system [9].

However, the burden and its consequences vary between caregivers, emphasizing the need for individualized and person-centered interventions [3]. It is therefore essential to assess evolving support needs. In addition, the Swedish government has asked for the possibility of exploring e-health solutions to improve support [10].

## Aim

The aim of this study is to describe the characteristics of informal caregivers in Sweden, their caregiver burden, psychological well-being and their support needs.

## Method

### Design

An online cross-sectional survey was used to collect data regarding characteristics, experiences, and support needs of informal caregivers in Sweden, as well as their perspectives on receiving support digitally through online platforms.

### Sample

Eligible participants were informal caregivers and individuals with informal caregiving experiences, aged 18 years or older, and mastered the Swedish language. Caregivers for children under the age of 18 were excluded as the regulations differ when it comes to children.

### Data collection and procedures

The data collection started on October 15, 2023, and ended on January 31, 2024. In order to collect data on the characteristics of informal caregivers (for example age, gender, education), the caregiver situation (for example, the number of people they care for, for how long they have provided care, how many hours, relationship to the care recipient and main reasons) and the need for support, questions were developed based on the Swedish National Carer Survey and a study conducted in Lithuania [2, 5, 13].

In addition, questions were asked about the use of technology and the willingness to receive digital support. To collect data regarding caregiver burden and psychological well-being, the survey also included the questionnaires Caregiver Burden Scale (CBS) [14], and the Depression, Anxiety, and Stress Scale (DASS-21) [15]. The survey was presented in Swedish, consisted of a total of 79 questions, and was estimated to take about 30–45 min to complete. See Supplementary document for an overview of the survey.

The survey was available online at iterapi.se, which is a secure platform using two-factor authentication for login and has been used in large numbers of studies [11, 12]. Information about the survey and a link to the platform was published on the Swedish Family Care Competence Centre, Carers Sweden, as well as other relevant carer and patient organizations. The information was also sent out to carer advocates and carer local organizations around the country, and published on social media (i.e., Facebook and Instagram) to reach out to informal caregivers. Interested informal caregivers registered their interest on the platform, reviewed the written information, and provided their consent to participate. Thereafter, they received an individual login (username and password) along with a request to change the password after the first login. The questionnaire could only be filled out once, but they had the opportunity to leave the survey if needed and go back and continue where they left off.

### Instruments

#### Caregiver burden

The CBS is a widely used tool to assess the impact of caregiving on individuals with dementia, renal dialysis and schizophrenia [16]. The instrument was developed and used to evaluate informal caregivers´ burden [17, 18], and has been validated in Swedish [19, 20].

CBS includes 22 items which can be presented as a total mean for the whole scale or divided into five different dimensions: general strain (8 items: no. 1, 3, 4, 5, 7, 10, 14, 19), isolation (3 items: no. 8, 12, 22), disappointment (5-items: no. 2, 13, 18, 20, 21), emotional impact (3 items: no. 6, 11, 16), and environment (3 items: no. 9, 15, 17). Each item is answered on a scale ranging between 1 and 4: “Not at all [1]” to “Yes to a large extent [4]”. The burden for the individual dimensions is presented as the percentage of responses within the following ranges: mean score within the range of 1.00-1.99 (low burden); 2.00-2.99 (moderate load) and 3.00-3.99 (high load). Previous reliability studies have shown high internal consistency for four of the five dimensions, with Cronbach’s α coefficients that varied between 0.70 and 0.87. However, the dimension ‘environment’ had a Cronbach’s α coefficient of 0.53 [14].

Cronbach α for the total scale in this study was 0.93 and for the subscales strain (α = 0.78), isolation (α = 0.85), disappointment (α = 0.74), emotional involvement (α = 0.83), and environment (α = 0.82).

#### Psychological well-being

Psychological well-being was measured using the DASS-21. The instrument has been evaluated in large clinical samples on patients with phobia and anxiety [21]. The evaluation has also been done in studies on psychological distress during covid-19 with a Cronbach α α = 0.98 for the total score, depression (α = 0.96), anxiety (α = 0.96) and stress (α = 0.98) [22]. DASS-21 has been translated into Swedish and includes 21 items divided into three sub-scales: depression, anxiety, and stress with seven items in each scale. Each item is scored on a four-grade Likert scale from “Did not apply to me at all (0)” to “Matched me very well [3]” [15]. Scores between 0 and 9 points indicate normal level of depression, 10–13 mild, 14–20 moderate, 21–27 severe and 28 and higher values very severe. For Anxiety, 0–7 points indicate normal level, 8–9 mild, 10–14 moderate, 15–19 severe, 20 and higher scores very serious. Corresponding values for Stress, 0–14 points mean normal stress, 15–18 mild, 19–25 moderate, 26–33 severe and 34 and higher scores very severe. In this study, Cronbach’s α were 0.91 for depression, 0.78 for anxiety, and 0.90 for stress.

### Data analysis

Data was analyzed using IBM SPSS Statistics V.29 (SPSS Inc., Chicago, IL, USA). To describe informal caregivers´ characteristics and care situation, categorical variables were presented as percentages and numbers or as means with standard deviations. One Way ANOVA with Tukey´s post hoc test or Student´s *t*-test were used to analyze the relationship between the caregiver burden, DASS-21 and the background variables (Table 1) and care situation (Table 2). Variables in Tables 1 and 2 were categorized into three or two options to facilitate the analysis. Regression analysis was performed to explore the associations between the variables from Tables 1 and 2, psychological well-being and caregiver burden. All related variables were entered in the analysis. *p* < 0.05 was set for statistical significance.

### Ethics

The study was approved by the Swedish ethical review authority (Etikprövningsmyndigheten) (Dnr. 2023/02843-01). All participants were informed and gave their consent on the first page of the survey to be able to answer the remaining questions. Respondents voluntarily participated and received detailed instructions on the homepage (iterapi.se) about the study and about the handling of their personal information in accordance with the EU data protection Act, GDPR (2001:99).

## Results

### Informal caregiver characteristics

The background questions were answered by 379 informal caregivers (Table 1). The majority (79%, *n* = 300) of those who responded were women and 82% (*n* = 309) were married or in a relationship. The age ranged between 18 and 89 years with a mean age of 61 years (± 16.7) for women and 62 years (± 15.9) for men. The vast majority (90%, *n* = 341) reported good financial status, 63% (*n* = 238) had a university degree, and almost half of them (46%) were still working. Of these 379, 332 (85%) answered questions about the caregiving situation, whereas 228 (60%) answered the questionnaires regarding caregiver burden and psychological well-being. A dropout analysis of the background variables presented in Table 1 showed that the only variable that significantly differed between those who answered the questionnaires and those who did not (*n* = 151, 40%) was those who had been informal caregivers for less than 12 months (73% vs. 55%, *p* = 0.020).

**Table 1:** Characteristics of informal caregivers

	All (*n* = 379)
**Age, years (m ± SD)**	62 ± 16.7
**Gender, n (%)**	
Women	300 (79)
Men	79 (21)
**Marital status, n (%)**	
In a relationship	309 (82)
Not in a relationship	70 (18)
**Economical situation, n (%)**	
Good	341 (90)
Problematic	38 (10)
**Educational level, n (%)**	
College/Higher vocational education/Upper secondary school	141(37)
University	238 (63)
**Occupational status, n (%)**	
Working	175 (46)
Not working	189 (50)
On sick leave/disability pension	15 (4)

### The caregiving situation

A total of 332 informal caregivers answered the questions regarding their caregiving situation, see Table 2. A majority (80%, *n* = 267) of the informal caregivers cared for one individual. Half (*n* = 161) provided care, help, and support for 1–10 h per day and 22% (*n* = 73) dedicated 60 h or more per week to caregiving responsibilities. As many as 88% (*n* = 292) had been informal caregivers for at least 12 months. Regarding the role as an informal caregiver, 65% (*n* = 214) cared for their spouse or children and 30% (*n* = 100) for their parents, siblings, or other relatives. Informal care was provided due to illness in 55% (*n* = 183) of the cases, and due to other reasons, such as age or disability in the rest of the cases (45%, *n* = 149).

**Table 2:** Caregiving situation

	All (*n* = 332)
**Number of individuals providing care and support to, n (%)**	
One person	267 (80)
Two persons	42 (13)
≥Three persons	23 (7)
**Duration of providing care, help and support, n (%)**	
Up to 6 months	21 (6)
Up to 12 months	19 (6)
≥ 12 months	292 (88)
**Average, daily care hours given (day and night), n (%)**	
0–10 h	161 (49)
11–59 h	98 (29)
≥ 60 h	73 (22)
**Relationship to care recipient, n (%)**	
Man/wife/partner/children	214 (65)
Parent/ Siblings/relatives	100 (30)
Guardian, neighbour, acquaintance	18 (5)
**The main reason for providing care, help and support, n (%)**	
Illness	183 (55)
Age	69 (21)
Disability	80 (24)
**Gender of the care recipient, n (%)**	
Woman	143 (43)
Man	183 (55)
Missing	7 (2)
**Age of the care recipient, n (%)**	
18–44	48 (14)
45–64 65>	47 (14) 236 (71)
Missing	1 (1)
**Living in same household, n (%)**	
Yes	165 (50)
No	167 (50)

Of those who received care, help and support from their caregivers, 56% (*n* = 183) were men and 71% (*n* = 236) were 65 years and older. 50% (*n* = 165) were living in the same household as the person they cared for.

### Informal caregivers’ burden and its relationship to demographic characteristics

**Fig. 1:**
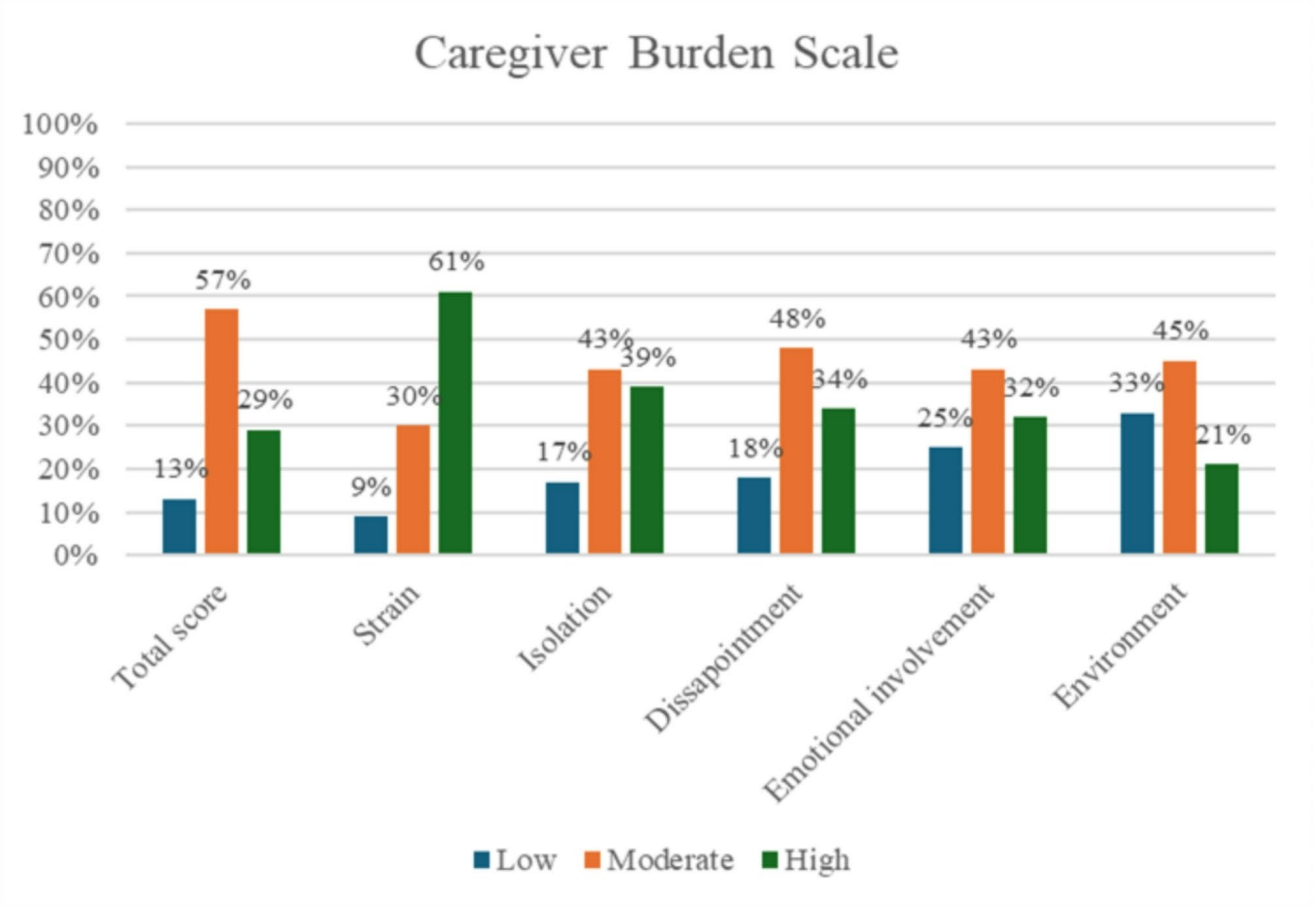
Distribution of informal caregivers according to caregiver burden scale, total score and subscales

A total of 228 informal caregivers responded to the CBS, of whom 86% (*n* = 200) scored moderate to high burden on the total scale. Based on the five different subscales, the analysis showed that the majority experienced moderate to high levels of general strain (90%, *n* = 207), isolation (82%, *n* = 189), disappointment (82%, *n* = 187), emotional impact (56%, *n* = 171) and environmental burden (66%, *n* = 151), see Fig. 1.

Table 3 shows the relationship between the caregiver burden and demographic characteristics. Regarding CBS total score, women scored higher mean values compared to men (2.72 ± 0.57 vs. 2.52 ± 0.66, *p* = 0.029). This was also found in the subscale general strain (24.73 ± 5.06 vs. 21.17 ± 6.20, *p* = 0.023). Caregivers on sick leave or disability pension scored higher on the subscale disappointment than those who were working (3.17 ± 0.58 vs. 2.53 ± 0.73, *p* = 0.029), but they did not differ compared to those not working (*p* = 0.653). Significant differences were also found in the subscale environment between those on sick leave or disability pension and those working (2.74 ± 0.59 vs. 2.09 ± 0.76, *p* = 0.040). Furthermore, informal caregivers with a problematic economic situation had higher mean scores on the subscale environment compared to those with a good economic situation (6.80 ± 2.12 vs. 5.64 ± 2.28, *p* = 0.004). A regression analysis showed that only the number of hours significantly impacted CBS (beta = 0.138, *p* = 0.038).

### Informal caregivers’ burden and its relationship to caregiving situation

The relationships between the caregiver burden and the caregiving situation are displayed in Table 4. Informal caregivers who cared for one person scored significantly higher on CBS total scale compared to those who cared for three persons or more (2.73 ± 0.56 vs. 2.35 ± 0.75, *p* = 0.038). The same relation was found in the subscale general strain (*p* = 0.017). After correcting for multiple testing (Tukey´s), the differences between the groups were no longer significant in the subscale’s disappointment and isolation.

**Table 3:** Relationships between the CBS total and subscales score, and the demographic characteristics of informal caregivers

	CBS total score		General strain		Isolation		Disappointment		Emotional involvement		Enviroment		
**Gender**		***p*** **= 0.029**		***p*** **= 0.023**		*p* = 0.801		*p* = 0.156		*p* = 0.899		*p* = 0.329	
Women Men	2.72 (0.57) 2.52 (0.66)		24.73 (5.06) 21.17 (6.20)		8.17 (2.57) 8.28 (2.97)		13.26 (3.59) 12.48 (3.45)		7.10 (2.23) 7.15 (2.40)		6.69 (2.22) 6.36 (2.08)		
**Civil status**		*p* = 0.993		*p* = 0.824		*p* = 0.298		*p* = 0.707		*p* = 0.303		*p* = 0.056	
In a relationship Not in a relationship	2.67 (0.55) 2.67 (0.71)		23.91(5.17) 23.69 (6.71)		8.29 (2.71) 7.88 (2.39)		13.03 (3.37) 13.26 (4.14)		7.18 (2.13) 6.76 (2.66)		6.41 (1.98) 7.21 (2.74)		
**Economic status**		*p* = 0.404		*p* = 0.329		*p* = 0.592		*p* = 0.053		*p* = 0.126		***p*** **= 0.004**	
Good Problematic	2.60 (0.54) 2.68 (0.60)		23.14 (5.19) 24.02 (5.61)		2.90 (0.93) 2.69 (0.86)		12.23 (2.94) 13.27 (3.65)		7.57 (2.18) 6.98 (2.27)		5.64 (2.28) 6.80 (2.12)		
**Occupational status**		*p* = 0.042		*p* = 0.053		*p* = 0.230		***p*** **= 0.015**		*p* = 0.321		***p*** **= 0.020**	
Working Not working On sick leave/ disability pension	2.59 (0.64) 2.85 (0.45) 3.09 (0.44)		2.92 (0.74) 3.29 (0.47) 3.40 (0.50)		2.55 (0.93) 2.80 (0.70) 3.40 (0.50)		2.53 (0.73) 2.90 (0.56) 3.17 (0.58)		2.38 (0.79) 2.02 (1.08) 2.51(0.88)		2.09 (0.76) 2,47 (0.70) 2.74 (0.59)		

**Table 4:** Relationships between the CBS total and subscales score, and caregiving situation

	CBS total score		General strain		Isolation		Disappointment		Emotional involvement		Enviroment	
**Number of individuals providing care and support to**		***p*** **= 0.007**		***p*** **= 0.004**		***p =*** **0.037**		***p*** **= 0.016**		*p* = 0.406		*p* = 0.382
One person Two persons ≥Three persons	2.73 (0.56) 2.46 (0.63) 2.35 (0.75)		3.05 (0.64) 2.75 (0.76) 2.56 (0.86)		2.80 (0.87) 2.51(0.84) 2.31(0.89)		2.68 (0.69) 2.38 (0.70) 2.27 (0.77)		2.39 (0.77) 2.21(0.65) 2.25 (0.74)		2.22 (0.73) 2.04 (0.65) 2.08 (0.84)	
**Duration of providing care, help and support**		*p* = 0.282		*p* = 0.290		*p* = 0.217		*p* = 0.177		*p* = 0.950		***p*** **= 0.046**
Up to 6 months Up to 12 months ≥ 12 months	2.54 (0.79) 2.42 (0.59) 2.69 (0.58)		2.81 (0.80) 2.71 (0.68) 3.0 (0.68)		2.47 (0.69) 2.36 (1.10) 2.76 (0.85)		2.30 (0.82) 2.42 (0.66) 2.64 (0.70)		2.33 (0.87) 2.43 (0.87) 2.36 (0.74)		2.50 (1.04) 1.73 (0.60) 2.20 (0.70)	
**Average, daily care hours given (day and night)**		***p <*** **0.001**		***p <*** **0.001**		***p <*** **0.001**		***p <*** **0.001**		*p* = 0.256		***p*** **= 0.024**
0–10 h 11–59 h ≥ 60 h	2.15 (0.76) 2.45 (0.58) 2.90 (0.57)		2.31(0.91) 2.76 (0.68) 3.19 (0.65)		1.90 (1.05) 2.33 (0.81) 3.11 (0.78)		2.24 (0.82) 2.37 (0.69) 2.91 (0.69)		2.30 (0.89) 2.19 (0.73) 2.41 (0.80)		1.66 (0.84) 2.16 (0.75) 2.37 (0.76)	
**Relationship to care recipient**		***p*** **= 0.016**		*p* = 0.244		***p <*** **0.001**		***p*** **= 0.002**		*p* = 0.288		*p* = 0.626
Partner/children Parent/Siblings Neighbour/acquaintance	2.78 (0.54) 2.77 (0.61) 2.54 (0.50)		3.06 (0.65) 3.12 (0.72) 2.91(0.57)		3.06 (0.78) 2.76 (0.80) 2.35 (0.80)		2.75 (0.67) 2.81(0.71) 2.42 (0.58)		2.45 (0.66) 2.25 (0.78) 2.31(0.82)		2.18 (0.63) 2.32 (0.83) 2.20 (0.76)	
**Gender of the care recipient**		***p*** **= 0.004**		***p*** **= 0.005**		***p*** **= 0.002**		***p*** **= 0.011**		*p* = 0.450		*p* = 0.087
Woman Man	2.55 (0.56) 2.78 (0.60)		22.79 (5.44) 24.84 (5.45)		7.61 (2.59) 8.67 (2.62)		2.48 (0.67) 2.72 (0.72)		6.99 (0.23) 7.21 (2.24)		6.34 (1.92) 6.83 (2.36)	
**Age of the care recipient**		*p* = 0.235		*p* = 0.061		*p* = 0.146		*p* = 0.346		*p* = 0.573		*p* = 0.356
18–44 45–64 ≥ 65	3.07 (0.38) 2.71 (0.65) 2.74 (0.75)		3.50 (0.37) 3.00 (0.76) 2.93 (0.85)		3.00 (0.80) 2.52 (0.73) 3.02 (0.93)		3.08 (0.42) 2.71(0.76) 2.79 (0.81)		2.66 (0.86) 2.35 (0.90) 2.43 (0.81)		2.40 (0.64) 2.49 (0.88) 2.15 (0.89)	
**Living in same household**		***p*** **= 0.002**		***p*** **= 0.016**		***p*** **< 0.001**		*p* = 0.011		***p*** **= 0.006**		*p* = 0.259
No Yes	2.55 (0.60) 2.79 (0.56)		23.00 (5.63) 24.75 (5.32)		7.31(2.58) 9.11(2.40)		12.50 (3.64) 13.68 (3.36)		6.69 (2.27) 7.50 (2.19)		6.66 (2.34) 6.52 (2.04)	

Informal caregivers who reported an average daily care of 60 h or more scored significantly higher on the CBS total scale than those who reported 0–10 h (2.90 ± 0.57 vs. 2.15 ± 0.76, *p* = 0.001) as well as 11–59 h (2.90 ± 0.57 vs. 2.45 ± 0.58, *p* < 0.001). The same results were found in the subscales general strain (*p* < 0.001 and *p* = 0.001), isolation (*p* < 0.001 and *p* < 0.001), disappointment (*p* = 0.017 and *p* < 0.001). Regarding environment, there was only a difference between those who reported 60 h or more and those who reported 0–10 h (*p* = 0.022).

Informal caregivers who cared for a partner or a child had higher scores on the CBS total scale compared to those who cared for a neighbor or an acquaintance (2.78 ± 0.54 vs. 2.54 ± 0.50, *p* < 0.014). This relation was also found in the subscale isolation (*p* < 0.01) and disappointment (*p* = 0.04). In addition, also those who cared for parents or siblings had higher disappointment scores (*p* = 0.020).

Women who cared for a male care recipient scored higher on the CBS total scale compared to men who cared for a female care recipient (2.78 ± 0.78 vs. 2.55 ± 0.57, *p* = 0.004). The same result was also found on the subscales general strain, isolation, and disappointment (*p* = 0.005; *p* = 0.002; *p* = 0.011). Living in the same household as the care recipient was also associated with higher scores on the CBS total scale (2.79 ± 0.56 vs. 2.55 ± 0.60, *p* = 0.002), as well as on the subscales general strain, isolation, disappointment, and emotional involvement (*p* = 0.016; *p* < 0.001; *p* = 0.011, *p* = 0.006).

### Psychological well-being

A total of 228 informal caregivers completed DASS-21. About 60% of them (*n* = 135, 59%) scored moderate to high levels of depression, 29% (*n* = 66) scored moderate to high level of anxiety, and 43% (*n* = 99) scored moderate to high levels of stress.

Regarding DASS-21 and demographic characteristics (Table 1), significant relationships were found between anxiety and working status. Caregivers who wasn`t working scored higher mean on anxiety (3.45 ± 3.04, *p* = 0.030) in comparison to those who were on sick leave or disability pension (2.44 ± 2.12, *p* = 0.548) and those who were working (1.70 ± 2.02, *p* = 0.030).

Concerning the relationships between DASS-21 and the caregiving situation, a significant association was found between depression and the gender of the care recipient. Respondents taking care of a man scored higher in comparison with those taking care of a woman (5.92 ± 3.62 vs. 4.59 ± 3.62, *p* = 0.012). The differences regarding anxiety were in the same direction but not statistically significant (2.11 ± 2.25 vs. 1.55 ± 2.11, *p* = 0.054).

Caregivers who were living in the same household had in comparison to those who were not living in the same household significantly higher mean values on depression (6.21 ± 3.94 vs. 4.44 ± 3.89, *p* < 0.001) and stress (7.46 ± 3.86 vs. 5.79 ± 4.40, *p* = 0,003).

**Fig. 2:**
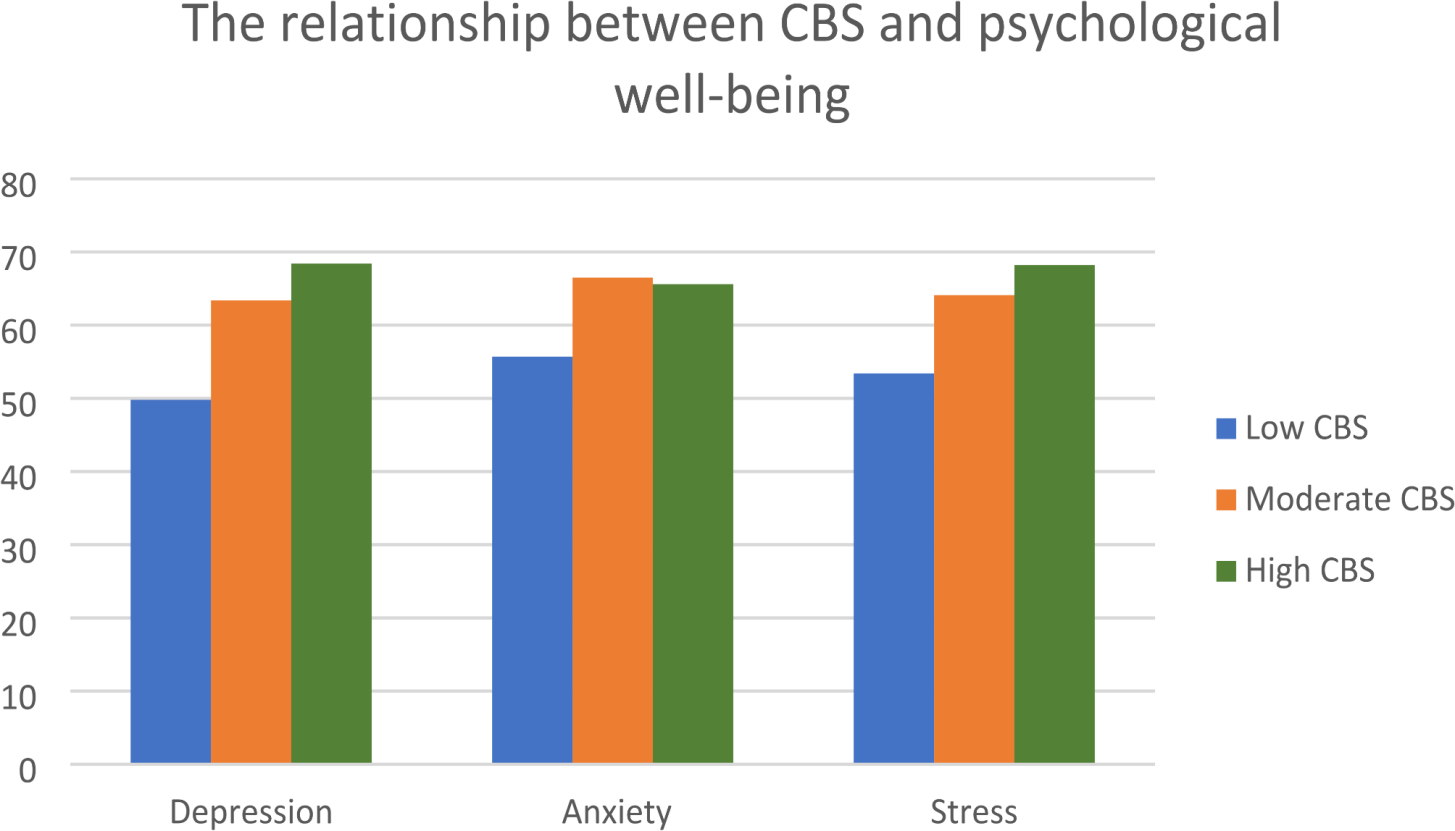
The relationship between CBS and psychological well-being

The relationship between caregiver burden, depression, anxiety and stress is shown in Fig. 2. A significant association was found between caregiver burden and depression (beta = 0.389, *p* < 0.001) and between caregiver burden and stress (beta = 0.274, *p* = 0.008).

### Support needs among informal caregivers

Regarding knowledge about existing support, approximately 50% were aware of the legal requirements and the support that the municipalities should offer informal caregivers. When rating the support, the top three forms of support requested by the informal caregivers were information and advice (38%), respite care (33%), and financial assistance (29%). The only significant correlation between these three prioritized support needs and demographic characteristics (as presented in Table 1) and caregiving situation (as presented in Table 2) was that the younger the care recipient, the more information and advice the informal caregivers desired (*r*=-0.34, *p* = 0.19). Concerning technology use in everyday life, 82% reported using technology every day, and 75% were interested in receiving support digitally (via websites and apps).

## Discussion

### Characteristics and challenges of informal caregivers in Sweden

In this study we aimed to describe the characteristics of informal caregivers in Sweden, their caregiver burden, psychological well-being and their support needs. We found that the majority of informal caregivers were women, many of whom possessed a good financial status and a university education. Most caregivers provided care for a single individual, typically a spouse or child, and nearly half of them were employed. Notably, one-fifth of the caregivers reported providing care for more than 60 h per week. A total of 86% reported a moderate to high caregiver burden on the total scale. Depression, anxiety, and stress were prevalent among the caregivers. The primary support needs identified were information and advice (38%), respite (33%), and financial assistance (29%), and 75% expressed interest in receiving the support digitally.

We found that over 80% of the informal caregivers scored moderate to high caregiver burden on the total scale of CBS, and between 66 and 91% reported moderate to high caregiver burden in the five dimensions (environment, emotional involvement, disappointment, isolation and strain), see Fig. 1. This suggests a high total caregiver burden among informal caregivers. This is in line with previous studies that show that informal caregivers who provides care to person with diseases such as dementia and depression in particular, experience a high total burden [23, 24].

### Caregiver burden: the impact of care hours, duration, and living arrangement

Informal caregivers in this study who reported an average week care of 60 h or more scored significantly higher caregiver burden than those who reported 0–10 h. This was also found in a study by Kirvalidze et al. (2023b) who also used the CBS scale and reported that spending more hours on care, help and support contributes to a high level of responsibility and demands, especially over a longer period, which can negatively affect mental health. Surprisingly we found that informal caregivers experience more burden when they care for one individual compared to if they care for several. A systematic review by Lindt, van Berkel [24] found that the duration of caregiving had a direct relationship with high caregiver burden, resulting in for example social isolation, difficulties in working, financial stress, and lack of freedom of choice. However, our results did not confirm this finding. In our study, informal caregivers living in the same household as the care recipient reported higher scores on the CBS total scale than those who did not live in the same household. According to Kirvalidze et al. (2023b) it can be described as a “role captivity” with feelings of overload, fatigue and being imprisoned, or trapped and losing oneself in one´s role as a caregiver [14]. This is prevalent in cases where informal caregivers are balancing care for both their children and aging parents, also called the “sandwich generation” [25].

### The psychological impact of caregiving: challenges and the need for support

Regarding the informal caregiver’s psychological well-being, moderate to high levels of depression, anxiety and stress were reported by 59%, 28% and 43% of informal caregivers respectively. It is known in previous studies that informal caregivers and particularly women experience a deterioration in psychological well-being at some point during their caregiving trajectory [1, 8, 26]. Being a woman and informal caregiver has a strong relationship with a high caregiver burden according to Lindt, van Berkel [24]. Thus, the caregiver burden and poor psychological well-being indicate negative consequences on multiple levels, mainly at the individual level on the caregivers health and life situation but also at the societal level, economic strains on welfare systems because of sick leave and part time work [4]. Another significant negative consequence is that caregiver’s own health problems, resulting from the caregiving burden, may turn them into consumers of health care system [9, 27]. This suggests a need for targeted support and interventions.

### Addressing the support needs of informal caregivers: insights and recommendations

According to this study, informal caregivers highlight a need for support. The three most common forms of support requested by the informal caregivers were information and advice (38%), respite (33%), and financial assistance (29%). Information and advice were prioritized as the most important need for support. This shows that the need for support among informal caregivers is not affected by their level of education. This emphasizes the importance of informal caregivers receiving support that is based on their conditions, resources and experience of their situation. Health literacy among informal caregivers is depending on how they feel in their role as informal caregivers in their situation or context [28, 29]. Several studies show that informal caregivers need support to reduce caregiver burden, depression, anxiety and stress [8, 30, 31]. Technology-based interventions, such as persuasive e-coaching application (PSD) which is including coaching to encourage and stimulate changes in users’ attitudes and behaviors by leveraging goal-setting and self-management components, have shown the potential to support informal caregivers [32]. This was also suggested in the National carers strategy in Sweden, where it is recommended to use welfare technology for example communication tools to contribute to increased independence of the informal caregivers for the public sector [10]. For such technology-based interventions, like PSD, interaction is suggested as it provides a chance to reflect on one’s emotions and situation with an external person. Having a place to express frustration might help reduce feelings of depression, anxiety and stress [32, 33]. The advantages of technology-based interventions include high flexibility and availability compared with traditional services, the limitation may include limited access to internet and technical equipment as well as technical competence [33, 34]. In a meta-analysis on the effectiveness of interventions that mitigate negative health outcomes in informal caregivers, it was found is that programs that are both multicomponent and person-centered seem to offer the greatest effectiveness and acceptability [35]. For further research it is therefore important to develop support programs that are person-centered, easily accessible and provide opportunities for reflection and expression, which can help reduce the caregiver burden, depression, anxiety and stress. Furthermore, this can help support and empower informal caregivers in their own situation and could ultimately also improve the informal care provided.

### Study weaknesses and strength

The study has several weaknesses and strengths. The cross-sectional design of this study makes it difficult to draw conclusions about causality, for example regarding whether caregiver burden causes psychological distress or the other way around. Challenges associated with online data collection include the potential exclusion of individuals with limited internet access, varying levels of digital literacy, and self-selection bias, where participants who choose to respond may differ systematically from those who do not. Additionally, online surveys may limit the ability to verify participant identity or ensure that responses are completed independently.

Another weakness is that participants in this study likely represent informal caregivers who are aware of the support available, as they were recruited through organizations and competence centers and may not be considered representative of all informal caregivers in Sweden. Furthermore, the majority of the informal caregivers in this study were women and our results therefor mainly indicate a caregiver burden based on women’s caring responsibilities. However, it is important to note that the majority of informal caregivers, not only in Swedish society but also internationally, are women, and the results of this study reflect this societal perspective. It is well known that it is more difficult to recruit men to this type of studies. In addition, the survey took 30–45 min to complete, which may have meant that informal caregivers with a heavy burden and the least time did not manage to complete the survey. This could mean that our results might risk underestimating the burden on informal caregivers. However, the dropout analysis showed that those who had been caregivers for the longest time (more than 12 months) were more likely to answer all the questions in the survey. Moreover, the results showed that the burden was linked to longer caregiving time. Thus, one can assume that our results mirror the situation of informal caregivers in general.

The strength of this study is that the informal caregivers who participated were those who identified themselves as such and actively sought knowledge about information and support. This indicates a high level of engagement and relevance, as the participants were motivated and self-selected. Another strength of the study is the flexibility of the digital survey on the study platform, which allowed participants to leave it incomplete and answer only the questions they wished to respond to. This flexibility ensured that the data collected was authentic and respected participants’ boundaries. Additionally, the survey’s digital format made it easily accessible on both computers and mobile phones, likely increasing participation rates and enabling a diverse range of caregivers to contribute, regardless of their location or access to specific technology.

From an ethical perspective, the study’s approach is also a strength. It was widely advertised on a national scale, reaching all municipalities and caregiver advocates in Sweden. Another strength was that the informal caregivers had the option to pause the survey when needed and resume it later, reducing the demands placed on them and making participation more manageable.

## Conclusion

Our results can have significant implications for understanding the challenges faced by informal caregivers. The study revealed that caregiving is often a long-term commitment, leading to moderate to high perceived caregiver burden and psychological well-being. The burden is particularly high when caring for one care recipient. The impact on burden and psychological well-being becomes more pronounced when the caregiver has a close relationship or lives in the same household as the care recipient. These findings underscore the urgent need for targeted support strategies and digital interventions to alleviate the burden on informal caregivers and enhance their psychological well-being. Therefore, future research should focus on developing and evaluating support strategies that align with caregivers’ preferences. This includes providing person-centered and easily accessible digital support. There is a need for larger longitudinal studies with more diverse populations to validate and expand upon these findings and investigate the long-term effects of informal care in relation to caregiver burden and psychological well-being.

## Electronic supplementary material

Below is the link to the electronic supplementary material.

Supplementary Material 1

## Data Availability

No datasets were generated or analysed during the current study.
