# Supplementary material for: Caregiver burden, psychological well-being, and support needs among Swedish informal caregivers
Source: BMC Public Health. 2025 Mar 4;25:867. doi: 10.1186/s12889-025-22074-y (PMC11881438; doi:10.1186/s12889-025-22074-y)
Supplement: Supplementary file 1 — Supplementary Material 1 [file 12889_2025_22074_MOESM1_ESM.docx]

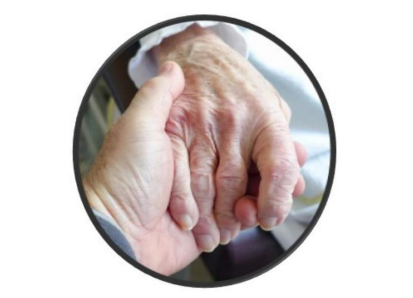


We would like to ask you if you would like to participate in a research project. This document will provide you with information about the project and what it means to participate.


**What is this project and why should I participate?**

This study is part of a research project where the main aim is to develop and evaluate an internet-based support program for informal caregivers in Sweden. With the help of a digital survey, we want to map your psychosocial health, find out what support you need and how you view receiving support digitally.

The survey is aimed at those who regularly provide care, help or support to a loved one (who is 18 years of age or older) due to physical or mental illness, disability or old age. By close we mean someone in your immediate or extended family, or someone else with whom you have a close relationship, such as a friend, neighbor or work colleague.

By "provide care, help or support" we mean things that you do on a regular basis, at least once a month. Think about all the care, help, and support you provide to family members or other people with whom you have a close relationship. It can be about personal care or supervision. It can also be more practical tasks such as shopping, transporting, taking care of the home or household, or dealing with authorities or healthcare professionals. It can also be helpful with managing finances, mail, bills and the like. 

This survey is not about the help, care and support you can provide in your profession, for example as a healthcare professional, personal assistant or guardian. The survey is only about the care, help and support you provide in your private life. 

**How does the study work?**

If you choose to participate in the study, it means that you fill out a questionnaire with a number of questions digitally. Filling out the survey is estimated to take about 30-45 minutes. By participating in the study, you will have the opportunity to make your needs as a family caregiver visible.

**What happens to my data?**

The information answered in the questionnaire will be stored in an encrypted database and stored for at least 10 years after the end of the study. Your answers will be treated confidentially, i.e. they will be assigned a code, and your identity will therefore not be revealed.

» Click here to learn more about how your data is handled.

**How do I get information about the results of the study?**

Contact the contact person for the study:

**Sonja Togmat Malki,** *Registered Nurse, Senior Lecturer/Doctoral Student***Tel:** 011-363325
**E-mail:** [sonja.togmat.malki@liu.se](mailto:sonja.togmat.malki@liu.se)

**Insurance and compensation**

No compensation will be paid. No additional insurance is required for this study.

**Participation is voluntary**

Your participation is voluntary, and you may choose to cancel your participation at any time. However, data already collected will be used. If you choose not to participate or want to cancel your participation, you do not need to give a reason, and it will not affect your situation as a family carer or you in your professional role.

If you wish to cancel your participation, please contact the principal investigator of the study (see below).

**Responsible for the study**

The principal investigator for the study is:


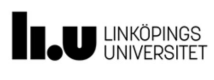
**Ghassan Mourad,** *Senior Lecturer, Associate* Professor
**Phone:** 011-363514
**E-mail:** [ghassan.mourad@liu.se](mailto:ghassan.mourad@liu.se)

| Age? |
| --- |
| _____ |

| What gender are you? By gender, we refer to gender identity, i.e. the gender you identify as. |
| --- |
| - Woman - Male - Non-binary - Other - Uncertain - Doesn’t want to answer |

| What is your marital status? |
| --- |
| - Married/Cohabiting - Widow/Widower - Divorced/Separated - In a relationship - Single - Other |

| What is your financial situation? |
| --- |
| - Very good - Good - Problematic - Very Problematic |

| What is your main occupation |
| --- |
| - Working - On sick leave / Disability - pensioner job seeker - Pensioner - Student - Housewife - Other |

| How many standard glasses do you drink in a typical week?  A standard glass of alcohol is equivalent to one of the following:  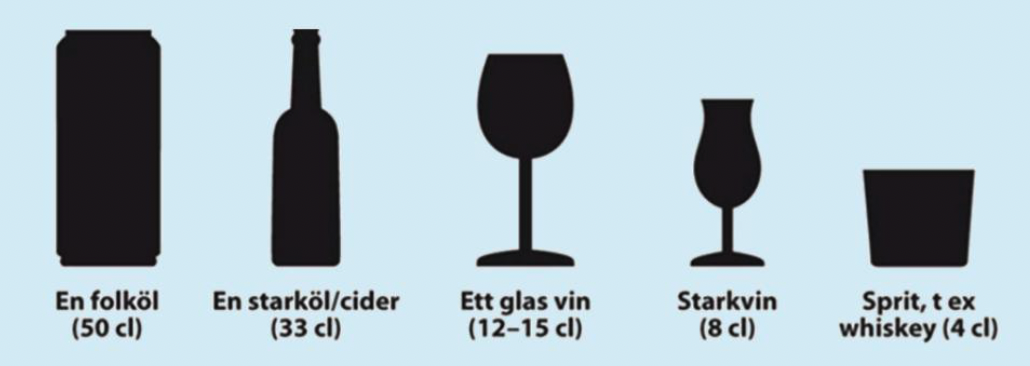  *For example: 50 cl of strong beer (5%) is equivalent to 1.5 standard glasses, a bottle of wine (75 cl) contains 6 standard glasses and a bottle of spirits (70 cl) contains 18 standard glasses.* |
| --- |
| - Drinking less than one standard glass per week or not at all - 1-4 standard glasses per week - 5-9 standard glasses per week - 10–14 standard glasses per week - 15 or more standard glasses per week |

| Have you used other drugs such as cannabis, amphetamines, cocaine or opiates, or have you used addictive drugs such as morphine or benzodiazepines without being prescribed by a doctor? |
| --- |
| - Yes - No |
| **If yes, how often?** |
| - 1 time/month or less often - 2-4 times/month - 2-3 times/week - 4 times/week or more |

**The situation as an informal caregiver**

| How many people do you provide care, help and support to? |
| --- |
| - One person - Two people - Three people or more |

| How long have you been providing care, help and support to the person you care for today? |
| --- |
| - Up to 6 months - Up to 12 months - 12 months or more |

| How many hours a week (day and night) do you provide care and support on average? |
| --- |
| - Less than 1 hour - 1-10 hours - 11-29 hours - 30-59 hours - 60 hours or more |

| The questions below are about the person you are providing care, support and help to. If you are helping more than one person, think only of the person to whom you provide the most care, support, and assistance when answering the questions.  What relationship do you have with the person to whom you provide care, support and help? |
| --- |
| - Spouse/husband/partner children - Parent - Sibling - Relative - Guardian, neighbor, acquaintance |

| What is the main reason why the person you are providing care, help and support to needs your help?  If you select "Other", also enter your choice in the provided text field. |
| --- |
| - Disease - Age - Disability - Other: _________________________ |

| What gender is the person you are providing care, support and help to? |
| --- |
| - Woman - Male - Non-binary - Other - Uncertain - Doesn’t want to answer |

| How old is the person you provide care, support and help to? |
| --- |
| - 18-29 - 30-44 - 45-64 - 65-79 - 80 years or older |

| Do you live in the same household as the person you are caring for? |
| --- |
| - 18-29 - 30-44 |

| Did you know before you received this survey that since 2009 there has been a provision in the Social Services Act (Chapter 5, Section 10) that means that relatives who care for and/or support informal caregivers must be offered support from the municipality? |
| --- |
| - Yes - No |

| Below is a list of different types of support for informal caregivers. State what form of support you want in your situation as a informal caregiver.  Rank which ones are most important to you (choose exactly 5 of these options, ranking from 1-5, with 1 being the most important) |
| --- |
| **A**. Information and Advice.  **B.** Training.  **C.** Counselling.  **D.** Support group for informal caregivers. **E.** Health-promoting activities such as walking, water aerobics, massage or similar.  **F.** Financial benefits or financial aid.  **G.** Respite care (temporary formal care for the person(s) you care for and support, to relieve you of responsibility for care, regardless of whether it is provided at home, through day care or in a home).  **H.** Healthcare facility.  **I.** Support via modern technology or the Internet (e.g. web information, video conferencing, Internet groups, GPS systems).  **J.** Support that facilitates work (possibility to work remotely, flexible working hours, communication with manager via the internet or similar).  **K.** Other, you can specify your preferred form of support below:   ____________________________________________________________________  1 ____ 2 ____ 3 ____ 4 ____ 5 ____ |

| What support do you have today as an informal caregiver? Pick the options that apply to you. |
| --- |
| - Information and advice - Information and advice Training - Counselling - Support group for informal caregivers - Health-promoting activities such as walking, water aerobics, massage or similar Health check-ups and health advice - Financial benefits or financial aid - Respite care (temporary formal care for the person or people you care for and support, to relieve you of responsibility for care, regardless of whether it is provided at home, through day care or in a home) - Healthcare facility - Support via modern technology or the Internet (e.g. web information, video conferencing, Internet groups, GPS systems) - Other, write here: _______________________ - No support today |

**Digital Support**

| How often do you use technology in |
| --- |
| - Every day - At least once a week - At least once a month |

| Would you consider using a digital support program as an informal caregiver?  A support program means a digital program that is available to help and support you as an informal caregiver. The program includes training and information as well as support aimed at helping you thrive and feel good in your situation as an informal caregiver. |
| --- |
| - Yes - No |

**The following questions are about the potential rewards provided by the care.**

**Please choose the answer option for each question that best matches your experiences.**

| 1. Not at all 2. A little 3. Sometimes 4. Quite a lot 5. Very much | 1 | 2 | 3 | 4 | 5 |
| --- | --- | --- | --- | --- | --- |
| 1. Does the care you give contribute to the sense that you are doing something important? |  |  |  |  |  |
| 2. Does the care that you are giving contribute to the fact that you are feeling satisfied with yourself? |  |  |  |  |  |
| 3. Is care rewarding by making life a little easier for your loved ones? |  |  |  |  |  |
| 4. Does care give you meaning to your life? |  |  |  |  |  |
| 5. Does care contribute to the sense that you are doing something meaningful? |  |  |  |  |  |
| 6. Is it rewarding for you to ‘just be there’ for him/her |  |  |  |  |  |
| 7. Does care provide you with personal growth? |  |  |  |  |  |
| 8. Are you happy that you are the one taking care of him/her |  |  |  |  |  |
| 9. Is the care rewarding because it makes your loved one happy? |  |  |  |  |  |
| 10. Is it rewarding to know that you are helpful to him/her? |  |  |  |  |  |

**The following questions are about how competent you feel as a carer.**

**Choose an answer that aligns with your experiences.**

**To what extent:**

| 1. Not at all 2. Little 3. Pretty much 4. Much | 1 | 2 | 3 | 4 |
| --- | --- | --- | --- | --- |
| 1. Do you think you have learned to handle very difficult situations? |  |  |  |  |
| 2. Do you feel that you are a good carer overall? |  |  |  |  |

**Now think about the daily ups and downs that you face as a carer: the work you do and the way you deal with difficulties.**

**If you weigh these things together:**

**To what extent:**

| 1. Not at all 2. Little 3. Pretty much 4. Much | 1 | 2 | 3 | 4 |
| --- | --- | --- | --- | --- |
| 1. Do you feel competent? |  |  |  |  |
| 2. Do you feel confident in yourself? |  |  |  |  |

**Please read each statement and choose the answer that best describes how the statement applies to you for**

**the past week. There are no right or wrong answers. Don't spend too much time on anything assertion.**

| 1. Didn't match me at all 2. Tuned in sometimes or part of the time 3. Tuned in quite a lot, or a lot of the time 4. Matched me very well or almost all the time | 1 | 2 | 3 | 4 |
| --- | --- | --- | --- | --- |
| 1. I found it difficult to unwind |  |  |  |  |
| 2. I felt like my mouth was dry |  |  |  |  |
| 3. I didn't seem to be able to feel any positive emotions at all |  |  |  |  |
| 4. I experienced breathing difficulties (e.g. excessively fast breathing even though I had not exerted myself physically)? |  |  |  |  |
| 5. I found it difficult to motivate myself to do things? |  |  |  |  |
| 6. I tended to overreact to events? |  |  |  |  |
| 7. I felt that I was shaking (e.g. my hands)? |  |  |  |  |
| 8. I felt that a lot of energy was spent worrying about me? |  |  |  |  |
| 9. I worried about situations where I could panic and make a fool of myself? |  |  |  |  |
| 10. It felt like I had nothing to look forward to? |  |  |  |  |
| 11. I noticed how I started to get upset |  |  |  |  |
| 12. I found it hard to relax |  |  |  |  |
| 13. I felt down and low |  |  |  |  |
| 14. I got annoyed by things that kept me from continuing with things I was doing |  |  |  |  |
| 15. I felt like I was about to panic |  |  |  |  |
| 16. I didn't manage to feel enthusiastic about anything |  |  |  |  |
| 17. I felt like I wasn't worth much as a human being |  |  |  |  |
| 18. I felt that I was quite easily offended |  |  |  |  |
| 19. I was aware of my heart rhythm even though I was not physically exerting myself (e.g., aware of my heart rate, or that my heart skipped a beat) |  |  |  |  |
| 20. I felt scared for no reasonable reason |  |  |  |  |
| 21. I felt that life was meaningless |  |  |  |  |

**Here are some questions regarding your situation as an informal caregiver.**

**Pick the option that suits you best. Give only one answer to each question.**

| 1. No, not all 2. No, barley 3. Yes, to some extent 4. Yes, to a great extent | 1 | 2 | 3 | 4 |
| --- | --- | --- | --- | --- |
| 1. Do you feel tired and overworked? |  |  |  |  |
| 2. Do you feel lonely and isolated because of your loved one's problems? |  |  |  |  |
| 3. Do you think you have to take on too much responsibility for your loved one's well-being and worries? |  |  |  |  |
| 4. Do you sometimes feel like you would like to run away from the whole situation you are in? |  |  |  |  |
| 5. Are you facing purely practical problems in healthcare that you find difficult to solve? |  |  |  |  |
| 6. Can you get hurt and angry with your relative? |  |  |  |  |
| 7. Do you think your health has suffered because you took care of your loved one? |  |  |  |  |
| 8. Have your loved one's problems led to reduced contact with others, such as family and friends? |  |  |  |  |
| 9. Is there anything in your relative's home that makes it difficult to take care of him or her? |  |  |  |  |
| 10. Do you feel bound by your loved one's problems? |  |  |  |  |
| 11. Do you feel disturbed by your loved one's behavior? |  |  |  |  |
| 12. Are your loved one's problems leading to you not being able to do what you had planned to do at this time in your life? |  |  |  |  |
| 13. Do you feel that it is physically stressful for you to take care of your relative? |  |  |  |  |
| 14. Do you think that your relative takes up so much time that you don`t get enough time for yourself? |  |  |  |  |
| 15. Are you worried that you won`t be able to take care of your loved one properly. |  |  |  |  |
| 16. Can you sometimes feel ashamed of your loved one's behavior? |  |  |  |  |
| 17. Is there anything in your surroundings around your relative's home that makes it difficult to take care of him or her? |  |  |  |  |
| 18. Has it meant any financial sacrifice for you to take care of your loved one? |  |  |  |  |
| 19. Do you find it mentally stressful for you to take care of your relative? |  |  |  |  |
| 20. Do you sometimes have the feeling that life has treated you unfairly? |  |  |  |  |
| 21. Had you thought that life would be different at the age you are now? |  |  |  |  |
| 22. Do you avoid inviting friends and acquaintances over because of your relative's problems? |  |  |  |  |
| **Here you have the option to add additional comments. Write down your own comments and thoughts. Your comments will be compiled with your other responses in this survey.** | | | | |
| ________________________________________________________________  ________________________________________________________________  ________________________________________________________________  ________________________________________________________________  ________________________________________________________________ | | | | |

| Your opinions and experiences are important. We plan to develop a support program in the next step. Do you want to be part of program? Fill in your contact details below and we will contact you for more |
| --- |
| ________________________________________________________________  ________________________________________________________________  ________________________________________________________________  ________________________________________________________________  ________________________________________________________________ |
